# Facial Expression Processing of Children Orphaned by Parental HIV/AIDS: A Cross-Sectional ERP Study with Rapid Serial Visual Presentation

**DOI:** 10.3390/ijerph18199995

**Published:** 2021-09-23

**Authors:** Qi Zhao, Hui He, Huang Gu, Junfeng Zhao, Peilian Chi, Xiaoming Li

**Affiliations:** 1 Department of Psychology, University of Macau, Macau 999078, China; yb97315@um.edu.mo (Q.Z.); peilianchi@um.edu.mo (P.C.); 2 Center for Cognitive and Brain Sciences, University of Macau, Macau 999078, China; 3 Institute of Behavior and Psychology, Department of Psychology, Henan University, Kaifeng 475004, China; hev499107@163.com (H.H.); XIAOMING@mailbox.sc.edu (X.L.); 4 Department of Health Promotion, Education, and Behavior, University of South Carolina, Columbia, SC 29208, USA

**Keywords:** children orphaned by parental HIV/AIDS, rapid serial visual presentation (RSVP), facial expression processing, event-related potentials

## Abstract

Existing behavioral studies have suggested that individuals with early life stress usually show abnormal emotional processing. However, limited event-related brain potentials (ERPs) evidence was available to explore the emotional processes in children orphaned by parental HIV/AIDS (“AIDS orphans”). The current study aims to investigate whether there are behavioral and neurological obstacles in the recognition of emotional faces in AIDS orphans and also to further explore the processing stage at which the difference in facial emotion recognition exists. A total of 81 AIDS orphans and 60 non-orphan children were recruited through the local communities and school systems in Henan, China. Participants completed a computer version of the rapid serial visual presentation (RSVP) task while recording ERPs. Behavioral results showed that orphans displayed higher response accuracy and shorter reaction time than the control (*p_s_* < 0.05). As for the ERPs analysis, the attenuated amplitude of N170 (i.e., an early component sensitive to facial configuration) was observed in AIDS orphans compared to the non-orphan control with happy and neutral faces; P300 (i.e., an endogenous component for affective valence evaluation in emotional processing) also showed significant differences in parietal lobe between groups, the non-orphan control group produced larger P300 amplitudes than orphans (*p* < 0.05). The results suggested that compared to the control group, AIDS orphans showed impaired facial emotion recognition ability with reduced brain activation.

## 1. Introduction

Children orphaned by parental Acquired Immunodeficiency Syndrome (AIDS) (“AIDS orphans”) are defined as children under 18 years of age who have lost one or both of their parents to HIV (The human immunodeficiency viruses) infection [1]. By 2019, the number of AIDS orphans worldwide reached 13.8 million children, most of whom were living in resource-poor environments (UNICEF, 2020). As established in the literature over the past decade, these AIDS orphans were exposed to numerous challenges, such as parental death, poverty, disrupted school attendance, and stigma [2,3,4,5,6]. All of these early life stress events have long-lasting effects on cognitive function and emotional response [7,8]. 

Existing behavioral research suggests that early life stress is linked to a greater affective reactivity [9]. Early life stress refers to children’s exposure to one or more events in childhood, which exceeds their coping ability and leads to long-term mental health and development problems. The childhood stressors usually include physical/sexual abuse, neglect, war or natural disasters, poverty, violence exposure, parental separation, parental illness or death, and drug abuse [10]. Individuals exposed to early life stress were more vulnerable to negative emotions, such as anxiety, loneliness, and depression [11,12], and also exhibited poorer emotion and cognitive outcomes, such as anger/hostility, attention problems, and aggressive behavior [13,14,15]. Recently, the underlying mechanism of these behavioral alterations was discussed in a substantial body of neurocognitive studies [16,17,18]. Neuroimaging studies showed that exposure to childhood life stress was associated with the aberrant development of emotional cognitive circuits, including the prefrontal cortex, amygdala, and hippocampus [19]. Event-related brain potentials (ERPs), with the higher temporal resolution, have been utilized to distinguish the neural sub-processes involved in behavior and reveal the impact of stress on the dynamic stage of emotional information processing [20]. However, empirical data were too limited to explore the emotional processes in AIDS orphans.

Facial expression, one of the most important emotional signals for humans that plays a crucial role in our daily lives, has often been used as emotion stimuli in ERP studies. Previous research has proposed the related ERP components of facial emotion processing [21,22]. Several ERP components, including N170, frontocentral N300, and P300 (the late positive potential), represent differential activity patterns in different face processing stages. The N170, a negative-going face-sensitive potential at occipital temporal sites which peaks at around 170 ms post-stimulus, is considered to be an index of the structural encoding of faces and a visual expertise stage of information processing [23,24]. As previous studies have demonstrated, N170 is clearly associated with an effect in the early phase of perception and attention processing [25,26]. N300 and P300 in the late-stage largely reflect the dimensionality of affective valence and stimulus evaluation [27,28,29,30]. Though these results and models were generalized using data from a healthy population, the neural basis on which early life stress impacts emotional expression processing still remains largely inconclusive.

Previous studies on AIDS orphans have mostly focused on hot issues such as resilience and mental health intervention and have paid little attention to the cognitive development characteristics and problems of these children [31,32,33]. One of our preliminary works indicated a deficit in the working memory process in AIDS orphans [34]. However, little is known about the social cognitive development of AIDS orphans. One study has shown that adolescents who have experienced persistent adversities showed different emotion recognition patterns from the control group, and these patterns may affect the children’s empathy for others [35]. Based on this evidence, the current study aimed to explore the behavioral and neurological obstacles in emotional faces processing in AIDS orphans. Specifically, besides response accuracy and time on behavioral tasks, the ERP technique was also applied to investigate whether there is a difference between AIDS orphans and non-orphan control group at the neural level. The present study further explored that whether the two groups differentiated in ERP components that sensitive to the emotional faces processing stage (i.e., N170 for the early phase of perception and attention processing, N300 and P300 in late-stage for affective valence and stimulus evaluation) in the rapid serial visual presentation (RSVP) paradigm.

## 2. Materials and Methods

### 2.1. Participants

Data were derived from a larger neurodevelopmental study in which a total of 90 children aged 9–17 years orphaned by parental AIDS (“orphans”) and 66 non-orphan children (“controls”) from the same grade levels were recruited through the local communities and school systems in rural central China [34,36]. As the recruitment procedure described in previous studies, the AIDS orphans were selected from a list of families and institutions caring for children affected by parental HIV in targeted communities. Children with a known diagnosis of HIV or AIDS were excluded from the larger study because of the potential impact of HIV infection or treatment on neurodevelopment. In this cross-sectional study, children (81 orphans and 60 controls) who had electroencephalogram (EEG) data were included. All of the children were right-handed, had a normal or correct-to-normal vision, reported no history of neurological diseases or injury, and had no structural brain abnormality. Before data collection, participants were made aware of the risks involved in the study and gave their informed consent. Specifically, written assent or oral assent (in case of illiteracy) was used for children. Appropriate written or oral permission (in case of illiteracy) was also obtained from caregivers/legal guardians who were available to provide consent for their children’s participation. Each participant received an age-appropriate gift at the completion of the experiment as a token of appreciation [23].

### 2.2. Stimuli and Procedure

The rapid serial visual presentation (RSVP) paradigm chosen for the present study can be used to investigate the characters of the time-based attention (Luo et al., 2010). The experimental procedure was programmed with E-Prime 2.0 (Psychology Software Tools, Inc., Pittsburgh, PA, USA). Materials consisted of 30 face pictures and 3 upright house pictures. Face pictures including 18 upright faces (6 happy faces, 6 fearful faces, 6 neutral faces) and 12 inverted neutral faces (male: female = 1:1), which were selected from the Chinese Facial Affective Picture System (CFAPS). Each subject completed a total of 108 trials. The task was presented in 2 blocks, and each block had 54 trials, with each facial expression being presented in 18 trials.

**Figure 1:**
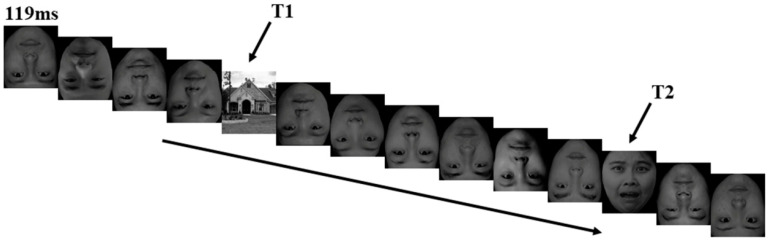
Overview of a representative experimental trial.

Participants were seated in a quiet and sound attenuated laboratory with their eyes approximately 100 cm from a 17-in monitor, which has a refresh rate of 60 Hz and a resolution of 1024 × 756 px^2^. Each trial began with a white fixation point (500 ms) and a blue fixation point (300 ms) appearing successively in the center of a black screen. Subsequently, a series of pictures composed of 2 target stimuli (T1 and T2) and 12 other pictures of distraction stimulus appeared rapidly with a stimulus onset asynchrony (SOA) of 119 ms and no blank inter-stimulus interval (ISI). The T1 stimulus was any of the three arranged house pictures; the occurrence probability of any of the three pictures was equal. The T2 stimulus was three positive faces. The distraction stimulus was 12 inverted neutral facial faces. T1 appeared randomly in the position of the third, fourth and fifth picture in a series of stimuli, while T2 appeared randomly in the position of the sixth picture after T1 stimulation (Figure 1). After the T1 and T2 stimuli, a 600 ms black blank screen appeared, followed by a question about T1 and a question about T2 continuously. Participants were required to make as accurate a judgment as they possibly could, and no time limit was set for their responses. Once participants responded by pressing a button, the question disappeared, and the next series would present after 500 ms of a blank screen. The subjects were allowed to take a short rest between the blocks. In order to eliminate the overlap of EEG before and after the fast presentation of stimulation and obtain pure T2-induced components, we set the corresponding baseline task according to Vogel et al. [37]. In the baseline task, the facial expressions at the T2 position were replaced by a black blank screen, and other conditions remained the same.

### 2.3. Apparatus

The EEG was recorded from 32 channels using the standard 10–20 system (Brain Products, Gilching, Germany) with a bandpass from 0.01 to 100 Hz and a 500 Hz sampling rate. All channels were online referenced to FCz during recording. Recording impedance for all electrodes was held beneath 10 kΩ.

### 2.4. ERP Data Preprocessing and Analysis

After data acquisition, EEG data were transferred into the EEGLAB and Letswave toolboxes, which are open-source MATLAB toolboxes for neurophysiologic data analysis. The EEG was offline, re-referenced to the average of the two mastoids, and filtered with a bandpass of 0.1–30 Hz. Epochs were extracted between the 200 ms pre-stimulus and 1000 ms post-stimulus time points, and the baseline correction was performed in the 200 ms pre-stimulus interval. The ocular artifacts of EEG data were removed by an offline method proposed by Gratton et al. [38]. After that, data were inspected and cleansed manually for any remaining identifiable artifacts, such as bad channels, eye blinks, and eye movements [23]. Eye movement artifacts and trials with EOG artifacts (e.g., a mean EOG voltage exceeding ± 80 μV) were automatically rejected. Especially, trials were accepted for analysis only if the response for T1 and T2 was correct. The averages were then digitally filtered (0.01–30 Hz, 24 dB/octave).

This study analyzed the ERP components N170, N300, and P300. The electrodes for further analysis were chosen according to ERP topographical distribution and previous studies. Specifically, the amplitudes and latencies of the N170 (200–280 ms) were analyzed at P7 and P8; the N300 (250–350 ms) at T7 and T8; and the P300 (450–650 ms) were analyzed at P3, Pz, and P4. According to the existing literature, the amplitudes of the N170 were measured as peak-to-peak values, while the amplitudes of the N300 and P300 were measured as mean values [23]. The time windows were determined through visual detection in the grand-averaged ERPs.

### 2.5. Statistical Analysis

SPSS 20.0 was used to perform a chi-square test to investigate whether the two groups (e.g., orphans and non-orphans control) differed on demographic factors (including age and gender). To compare the behavioral response performance between two groups, ANCOVA was conducted with the accuracy and response time as a dependent factor, group as an independent factor, and age as a covariate. The accuracy was calculated as the percentage of correct responses of all trials, the response time was calculated as the mean response time of all correct trials. A repeated-measures ANOVA was conducted on behavioral and ERP data with electrode point (N170: P7 and P8; N300: T7 and T8; P300: P3, Pz, and P4), facial emotion (happiness, neutral, and fear) as within-subject factors, and participant group (orphans vs. control group) as a between-subject factor. The Kolmogorov-Smirnov test was conducted to assess the normality of the distribution before conducting an ANOVA. For all the analyses in this study, *p* < 0.05 was considered to be statistically significant, and the *p*-values were adjusted using the Greenhouse-Geisser correction when appropriate [23].

## 3. Results

A significant difference in age was showed between the orphans (14.64 years of age) and the control group (12.43 years; *t* = 87.372, *p* < 0.001). Therefore, age was used as a covariate in the subsequent analyses. Other demographic variables did not show any significant difference between groups (all *p* > 0.05, Table 1).

### 3.1. Behavioral Performance

ANCOVA results revealed a significant main effect of group (F_1__, 137_ = 9.674, *p* = 0.002, η^2^ = 0.066) on the response accuracy, with orphans displaying a higher accuracy (87.30 ± 11.09%) than the control (83.88 ± 13.01%). The repeated-measures ANOVA result revealed that response accuracy was significantly affected by facial emotion (F_2, 276_ = 6.756, *p* = 0.003, η^2^ = 0.047) while age was a covariate. Pairwise comparison results showed that the accuracy of neutral faces (89.18 ± 11.87%) was higher than that of happy (85.18 ± 16.93%, *p* < 0.001) and fearful faces (86.11 ± 15.58%, *p* = 0.002).

ANCOVA results of the response time also showed a significant difference in the two groups (F_1__, 137_ = 4.539, *p* = 0.035, η^2^ = 0.032), the response time of orphans (745.05 ± 256.72) was shorter than that of the control (904.32 ± 318.92). The repeated-measures ANOVA results of the response time showed no significant difference in all conditions (all *p_s_* > 0.05).

### 3.2. ERP Data Analysis

#### 3.2.1. N170

No significant main effect of facial emotion or participant group was shown on the N170 amplitudes at P7 and P8 (Table 2). However, there was a significant interaction effect of facial emotion × participant group × electrode point (F_2, 276_ = 3.889, *p* = 0.028, η^2^ = 0.027), demonstrating that N170 amplitudes were affected by task category and this category-specific modulation was more pronounced over the right hemisphere (electrode P8). 

The results at P7 showed no difference between participant groups and facial emotion in all conditions (*p* > 0.05). N170 amplitudes at P8 showed significant difference on facial emotion (F_2, 276_ = 3.907, *p* = 0.033, η^2^ = 0.028). Neutral faces (−2.838 μV) elicited smaller amplitudes than happy (−3.894 μV) and fearful faces (−4.673 μV). The difference between participant groups indicated a trend-level significant effect (F_1, 138_ = 3.613, *p* = 0.059, η^2^ = 0.026), thus we conducted further analysis for each group separately. The results showed that there was a significant difference across the three facial emotions in the orphan group (F_2, 158_ = 5.448, *p* = 0.016, η^2^ = 0.065), while no significant difference was found in the control group. 

#### 3.2.2. N300

N300 amplitudes at T7 and T8 revealed an effect on the facial emotion (F_2, 276_ = 3.206, *p* = 0.044, η^2^ = 0.023), and on the interaction between facial emotion and participant group (F_2, 276_ = 3.537, *p* = 0.032, η^2^ = 0.025). Further analysis at T7 did not show any significant effect, while N300 amplitudes at T8 showed significant difference across the three facial emotions (F_2, 276_ = 3.590, *p* = 0.030, η^2^ = 0.025), and significant interaction effect of facial emotion × participant group (F_2, 276_ = 4.536, *p* = 0.012, η^2^ = 0.032). 

The simple effect analysis at T8 showed a significant difference across the three facial emotions (F_2, 158_ = 4.804, *p* = 0.011, η^2^ = 0.057) in the orphan group but not in the control group (*p* > 0.05). Neutral faces (3.305 μV) elicited smaller amplitudes than happy (2.765 μV) and fearful faces (1.771 μV) in the orphan group, while the neutral faces (1.315 μV) elicited larger amplitudes than happy (2.516 μV) and fearful faces (1.925 μV) in the control group.

#### 3.2.3. P300

**Figure 2:**
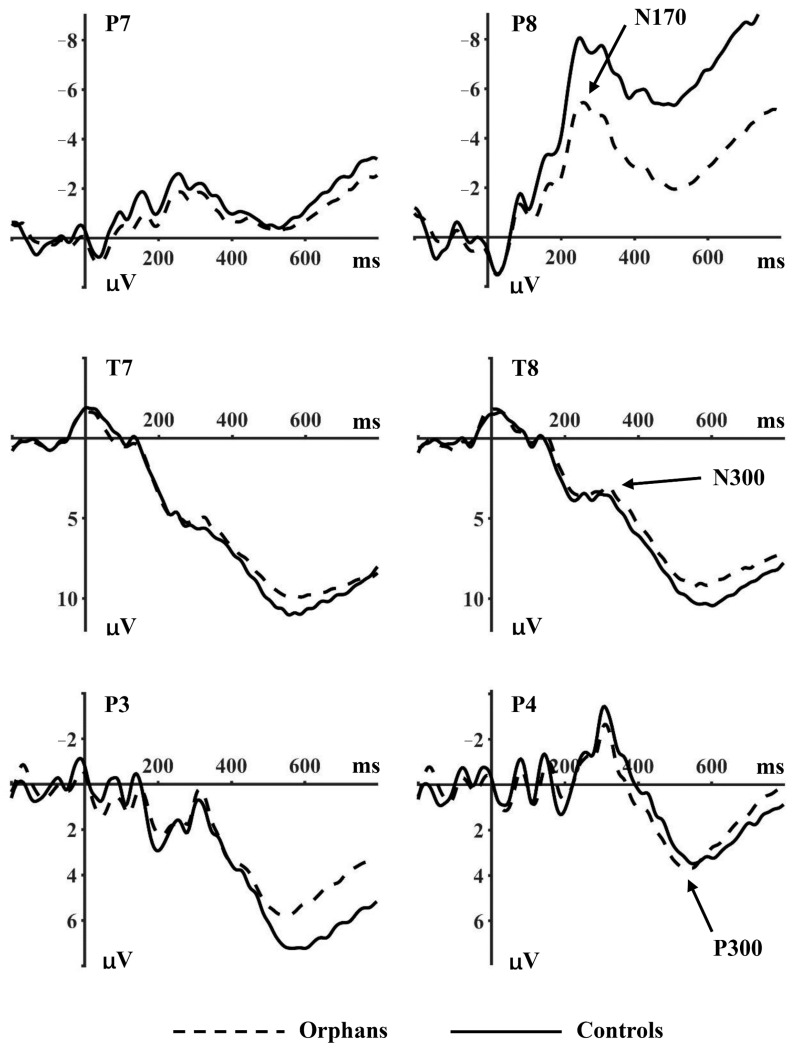
Grand average ERPs for orphans and control group.

As shown in Figure 2, there were significant differences between the participant group (F_1, 138_ = 8.926, *p* = 0.003, η^2^ = 0.061), facial emotion (F_2, 276_ = 3.155, *p* = 0.045, η^2^ = 0.022) and electrode point (F_2, 276_ = 6.017, *p* = 0.003, η^2^ = 0.042) on P300 amplitudes at P3, Pz, and P4. The control group elicited larger amplitudes (9.323 μV) than the orphan group (4.222 μV). The results of further analysis at each electrode point revealed a significant interaction between participant group and facial emotion (P3: F_2, 276_ = 4.670, *p* = 0.012, η^2^ = 0.033; Pz: F_2, 276_ = 3917, *p* = 0.027, η^2^ = 0.028; P4: F_2, 276_ = 4.213, *p* = 0.017, η^2^ = 0.030).

The simple analysis showed a significant between-group difference in happy (F_1, 138_ = 15.196, *p* < 0.001, η^2^ = 0.099) and fearful faces (F_1, 138_ = 7.822, *p* = 0.006, η^2^ = 0.054). The control group elicited larger amplitudes of happy (10.492 μV) and fearful faces (10.554 μV) than the orphan group (3.560 μV; 3.680 μV), while there was no significant effect of neutral faces. Further analysis at P3 showed a significant difference on facial emotion in the control group (F_2, 118_ = 3.507, *p* = 0.034, η^2^ = 0.056), while there was no significant difference in the orphan group.

## 4. Discussion

The primary goal of this study was to examine the effect of early life stress events, specifically parental death of HIV/AIDS, on children’s processing of facial displays of emotion. Though results showed that AIDS orphans worse in detecting facial emotions than non-orphan control children at the behavioral level, the ERP results showed evidence of abnormal neural responses to emotional stimuli during facial emotion processing in AIDS orphans. 

The first key finding was the attenuated N170 amplitude in AIDS orphans compared to the non-orphan control group with happy and neutral faces at the P8 electrode, whereas no difference was observed with fear faces between groups. This result was in accordance with existing evidence that similar activity alterations during emotional face processing were found in individuals with a hearing disability, anorexia nervosa, attention-deficit/hyperactivity disorder, or schizophrenia [23]. The finding might reflect the difficulty in structural processing of the face in AIDS orphans. Moreover, this result supports the notion that the N170 sensitivity is heterogeneous. According to previous studies, N170 was affected by emotional expression in general but did not discriminate between different emotions [30,39,40,41]. In the present study, N170 differed by emotional expression, with larger N170 amplitude elicited by happy and fearful facial expressions than neutral faces. However, the happy expressions did not differentiate between fearful expressions. It suggested that N170 was sensitive to emotional faces but did not discriminate between diverse emotional expressions. Meanwhile, AIDS orphans showed diminished N170 amplitude during the processing of happy and neutral faces but not during the processing of fearful faces. Estes and Verges [42] found individual responses to emotionally negative stimuli to be more automatic relative to positive or neutral stimuli. Therefore, the unhindered processing of negative stimuli here (e.g., the fearful expression) might indicate a processing advantage of the threatening signal during strained attention conditions (e.g., among AIDS orphans). 

The results of the current study also revealed a different P300 activation pattern between AIDS orphans and the non-orphan control group. Compared to the non-orphan control group, AIDS orphans were less prone to process happy and fearful faces. Moreover, the P300 component differentiated between the three types of emotional faces in the control group, but this difference was absent in AIDS orphans. The heterogeneous phenomenon demonstrated in the control group is consistent with the hypothesis of three stages of facial expressions processing proposed by Luo et al. [30], depicting that various emotional facial expressions were distinguished as positive or negative at later processing stages. Although the neural mechanism studies of AIDS orphans were data deficient, many ERP studies in ADHD children, deaf children, children of addicted parents, and patients with depression and schizophrenia have indicated less sustained attention to emotional stimuli and the absence of heterogeneous during P300 [23,43,44,45,46]. The P300 component, an index of conscious and elaborate evaluation of emotional stimuli [47], is also thought to be associated with the assignment of emotional meaning (semantic value) to faces [48,49]. Therefore, one possible explanation for the finding could be that compared with the control group, AIDS orphans may be more likely to assign sufficient emotional semantic meanings to happy and fear stimuli during facial emotion processing. 

Our findings suggested that compared to the non-orphan control group, the AIDS orphans showed impaired facial emotion recognition ability with reduced brain activation. Although AIDS orphans were not doing worse in detecting facial emotions than control children at a behavioral level, the ERP results showed evidence of abnormal neural responses to emotional stimuli during facial emotion processing in AIDS orphans. 

Finally, several limitations of the current study should be mentioned. First, although efforts were made in this study to match the orphan and control group by grade, there was still a significant age difference between the two groups. Thus, future research may increase the number of participants and verify the results based on an age-matched control group. Second, based on the current results of the ERP experiment, we mainly observed a difference in the time process of emotional processing between orphans and the control group. In future studies, we may conduct fMRI research to explain the spatial difference of brain mechanisms further. Moreover, the findings here were based on the cross-sectional data with a relatively small sample size; there is also a need to collect longitudinal data in the future to explore the long-term development of emotional cognition and investigate the validity and applicability of our findings in a larger sample of AIDS orphans and other children with early adversity experiences. Although our research has obvious limitations and the external validity of our research is uncertain, under the background of public health, it is an important discovery to show the abnormal neural activities of AIDS orphans, which deserves us to further explore the cognitive neural activity of this group of people and other children who experience early life stress, and provide a theoretical basis for education and nursing institutions to carry out more effective and targeted interventions and activities. 

## 5. Conclusions

The current study investigated the impact of early life stress events on children’s processing of facial displays of emotion. According to the results, AIDS orphans may have difficulty in structural processing of the face, especially with happy and neutral faces. They also showed a worsened evaluation ability of information related to the affective valence of a face. However, negative stimuli were given more weight during strained attention conditions. Overall, the findings indicate that early life adversity may result in worse emotional outcomes among AIDS orphans through impaired configuration and evaluation processing.

## Figures and Tables

**Table 1:** Demographic variables and behavioral performance between groups.

		Orphan Group	Control Group	F or χ^2^	*p*
Number of subjects		81	60		
Gender *N*(%)	Boys	46 (65.7%)	24 (34.3%)	3.887	0.061
	Girls	35 (49.3%)	36 (50.7%)		
age (years)		14.64 ± 1.43	12.43 ± 1.62	87.372	<0.001
Accuracy (%)		0.87 ± 0.11	0.84 ± 0.13	9.674	0.002
Response time (ms)		745.05 ± 256.72	904.32 ± 318.92	4.539	0.035

Note. Values are presented as mean ± standard deviation (SD).

**Table 2:** The average amplitudes (μV) of the ERP components between groups.

ERP Components	Facial Emotion	Electrode Point	Average Amplitudes (μV)		F or χ^2^	*p*
			Orphans	Controls		
N170	Happy	P7	−2.09 ± 3.94	−1.75 ± 3.66	0.261	0.610
		P8	−2.86 ± 8.60	−5.29 ± 4.02	4.135	0.044
	Neutral	P7	−1.71 ± 9.13	−1.67 ± 7.35	0.001	0.978
		P8	−1.87 ± 5.23	−4.15 ± 6.23	5.548	0.020
	Fearful	P7	−1.45 ± 6.32	−2.55 ± 5.87	1.094	0.298
		P8	−4.25 ± 8.14	−5.24 ± 4.77	0.702	0.404
N300	Happy	T7	3.28 ± 5.20	4.33 ± 4.32	1.606	0.207
		T8	2.76 ± 4.42	2.52 ± 3.95	0.119	0.730
	Neutral	T7	3.50 ± 5.39	2.25 ± 8.28	1.177	0.280
		T8	3.30 ± 5.93	1.31 ± 6.72	3.466	0.065
	Fearful	T7	2.49 ± 7.79	2.72 ± 6.80	0.032	0.858
		T8	1.77 ± 6.24	1.93 ± 3.96	0.028	0.867
P300	Happy	P3	5.09 ± 10.44	8.16 ± 8.87	3.379	0.068
		P4	3.29 ± 10.53	5.01 ± 9.62	0.988	0.322
		Pz	8.57 ± 9.90	9.83 ± 7.86	0.669	0.415
	Neutral	P3	5.60 ± 8.92	5.41 ± 10.80	0.013	0.909
		P4	4.69 ± 7.55	3.41 ± 10.94	0.679	0.411
		Pz	9.69 ± 10.09	6.94 ± 10.76	2.436	0.121
	Fearful	P3	6.18 ± 13.11	7.59 ± 11.14	0.455	0.501
		P4	4.33 ± 10.59	4.96 ± 11.67	0.122	0.739
		Pz	8.13 ± 18.17	8.87 ± 10.97	0.079	0.778

Note. Values are presented as mean ± standard deviation (SD).

## Data Availability

The data that support the findings of this study are available on request from the corresponding author. The data are not publicly available due to privacy or ethical restrictions.
